# Case report: Induction and maintenance of steroid-free remission with vedolizumab in a case of steroid-dependent autoimmune pancreatitis

**DOI:** 10.3389/fimmu.2023.1201363

**Published:** 2023-06-19

**Authors:** Paul Griebel, Florian Tran, Janina Luehring, Stefan Schreiber

**Affiliations:** ^1^ Institute of Clinical Molecular Biology, University Medical Center Schleswig-Holstein, Kiel, Germany; ^2^ Department of Internal Medicine I, University Medical Center Schleswig-Holstein, Kiel, Germany

**Keywords:** case report, gastroenterology, pancreatitis, autoimmune pancreatitis, vedolizumab, autoimmunity

## Abstract

Autoimmune pancreatitis responds well to corticosteroids in most instances. Additional immunosuppression or low-dose maintenance steroids may be necessary upon relapse. There is limited data on alternative strategies when these regiments fail or cause adverse reactions. We report a case of a middle-aged woman with autoimmune pancreatitis in whom tapering of prednisolone below the dose of 25mg per day resulted in relapse of symptoms and long-term steroid use led to development of steroid induced hyperglycaemia. Induction and maintenance of steroid-free remission was ultimately successful under vedolizumab therapy. Remission has been stable for over one year with reduced need for antidiabetic intervention. This is the first reported case of treatment of refractory autoimmune pancreatitis with vedolizumab. It highlights the overlap of immunological mechanisms within inflammatory diseases of the digestive tract and how knowledge of biological data can inform treatment decisions for individual cases. The demonstrated efficacy of vedolizumab and low risk of severe side effects warrant further investigation into its use in autoimmune pancreatitis.

## 1 Introduction

Autoimmune pancreatitis (AIP) is a rare subtype of chronic pancreatitis, associated with cellular and humoral self-reactivity (1). Diagnostic criteria include typical symptoms, findings on imaging and response to steroids. The disease can be divided into two types: Type 1 AIP is strongly associated with increased immunoglobulin G, subclass 4 (IgG4) serum levels and concomitant IgG4-related diseases. Type 2 AIP is considered to be dependent on cellular immune responses involving CD4+ T cells, and often coincides with inflammatory bowel disease (IBD) (2–4).

Treatment consists of high dose corticosteroid regimen followed by a period of steroid tapering. In case of relapse, low-dose maintenance therapy with steroids, alternative immunosuppressive agents such as azathioprine or anti CD20 therapy with Rituximab (RTX) can be considered (5, 6). Since AIP is a rare disease, there is limited data for potential alternatives in case of treatment failure or adverse reactions.

## 2 Case report

A woman in her early 60s was admitted to the hospital by her primary care physician with rapid weight loss of 12kg within three months and exsiccosis associated with steatorrhea. She had experienced unspecific abdominal discomfort within the last months. The past medical history was notable for metabolic syndrome, hypothyroidism, and depression. Her type 2 diabetes was well controlled with empagliflozin (10 mg/day).

**Figure 1:**
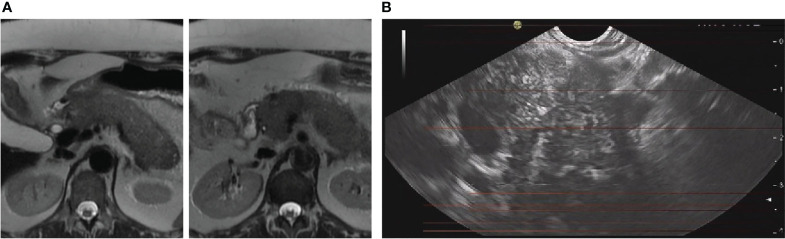
Single-shot fast spin-echo MRI sequence showing diffuse inflammatory infiltration of the pancreas **(A)**. Endosonographic image showing massive hypoechoic swelling, multiple interlobular septa, and diameter changes **(B)**.

An abdominal MRI ordered by the patient’s general practitioner was performed two weeks before presentation and revealed diffuse inflammatory infiltration of the pancreas, without exudates, necrosis, or focal lesions (**Figure 1**). She was transferred to the clinic for endoscopic workup. Endoscopic ultrasonography showed generalized swelling and increased echogenicity of interlobular septa (**Figure 1**). Fine-needle biopsy revealed unspecific chronic inflammatory infiltrates without signs of fibrosis or malignancy. Laboratory tests were unremarkable, without elevation of C-reactive protein or lipase. IgG4 was within normal limits. These findings were most consistent with the diagnosis of probable type 2 autoimmune pancreatitis. Further workup included esophagogastroduodenoscopy and colonoscopy with multiple biopsies and methylene blue staining, to rule out inflammatory processes in the upper intestinal tract that could involve the pancreatic duct or tissue. There were no signs for malignancy, Coeliac’s disease or Whipple’s disease. The examination also revealed no underlying IBD. Anti-tissue transglutaminase immunoglobulins A and G, as well as carbohydrate antigen 19-9 levels were within normal limits.

Although the histology was non-conclusive for autoimmune pancreatitis, the clinical symptoms in combination with typical imaging findings warranted treatment initiation with prednisolone (50 mg/day) and substitution of pancreatic enzymes to treat steatorrhea. Symptoms resolved under steroid-therapy, which is another hallmark of AIP.

**Figure 2:**
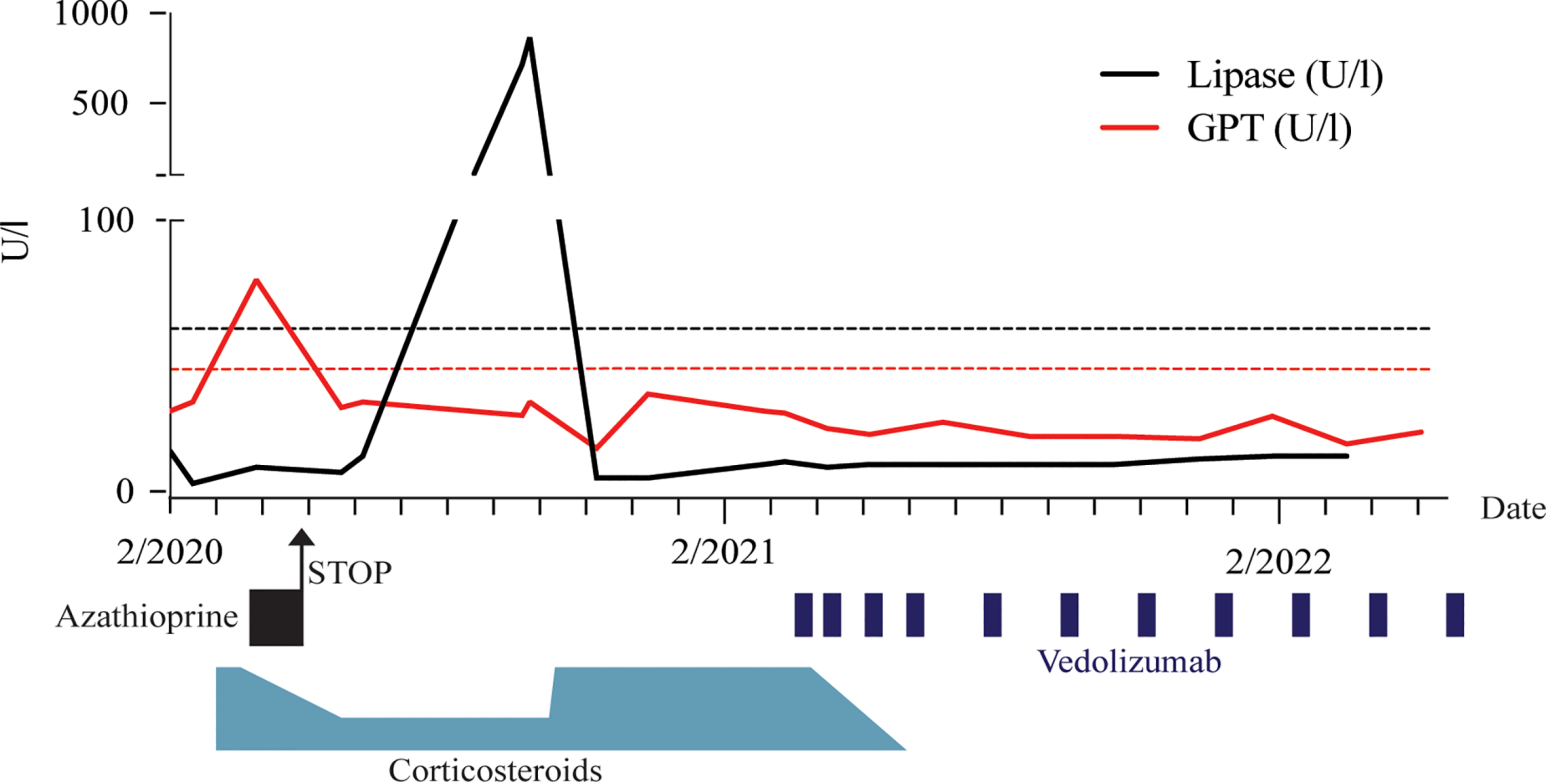
Timeline of immunosuppressive treatment regimen and associated glutamate pyruvate transaminase (GPT) and lipase levels. Dashed lines indicate respective upper limits.

The initial taper quickly resulted in reoccurrence of gastrointestinal symptoms, specifically nausea and diarrhoea. We responded by adding supportive immunosuppression through azathioprine, which eventually had to be discontinued due to a rise in liver enzymes (**Figure 2**) as well as abdominal pain and nausea. Despite this, steroids could be kept at a low level and even further tapered over the next 6 months. Unfortunately, the patient then had another flare of steatorrhea associated with a dramatic increase in lipase (**Figure 2**). This prompted a return to high-dose steroid treatment, which could no longer be decreased below 25mg/day without recurrence of symptoms. Under this high dose glucocorticoid regimen, the patient’s type 2 diabetes therapy had to be escalated to include insulin. Because of the ongoing pandemic and concerns regarding the use of Rituximab and concomitant vaccination, we considered available data on a potential alternative for treatment escalation (7).

Vedolizumab is a biological agent which inhibits proinflammatory lymphocyte migration into tissue through binding of α_4_β_7_ integrin to mucosal vascular addressin cell adhesion molecule 1 (MAdCAM-1) on high endothelial venules of the gut. This therapeutic principle is utilized in the targeted treatment of chronic intestinal inflammation, as found in IBD, intestinal graft-versus-host disease and autoimmune colitis after immune checkpoint inhibition. In addition to the expression in intestinal tissue, MAdCAM-1 has been shown to be expressed in high endothelial venules within chronically inflamed pancreatic tissue (8, 9).

Given this expression data, the association of IBD with AIP, the efficacy of anti-integrin therapy as well as its favourable safety profile (10), α_4_β_7_ integrin blockade appeared to be an interesting mode of action in this case. We also considered tumor necrosis factor-α (TNF-α) inhibition, which is more commonly used for IBD, but there were concerns regarding vaccine efficacy and its role in viral defense (11–13).

Thus, a multi-disciplinary board for inflammatory diseases in consultation with the patient and her medical insurance, decided upon an off-label trial with vedolizumab after reviewing the literature, aiming for a successful weaning from corticosteroids.

The chosen vedolizumab treatment regimen was identical as in IBD: In the induction phase, 300mg vedolizumab were administered IV at weeks 0, 2 and 6, followed by 300mg vedolizumab IV every 8 weeks as a maintenance therapy starting from week 14.

The vedolizumab infusions were well tolerated without occurrence of adverse events except for fatigue and headaches for two days after each infusion, both common and known side effects (10). Starting from 30 mg/day the steroid dose could be increasingly tapered following the first administration of vedolizumab. After 6 weeks, upon receiving her third dose, the patient was well maintained with 5 mg prednisolone per day. After a physiological response to adrenocorticotropic hormone (ACTH) was established, steroids could be discontinued entirely.

**Figure 3:**
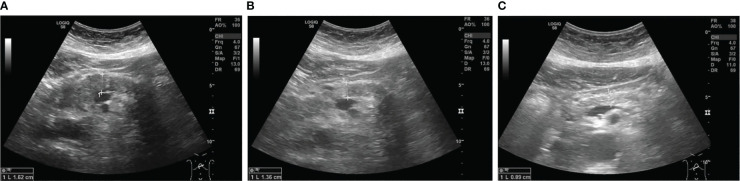
Sonographic images of the pancreatic body showing diffuse hypoechoic swelling upon admission **(A)**, which decreased under steroid treatment **(B)** and 11 months after the first dose of vedolizumab **(C)**.

Within the follow-up period of one year there has been no relapse of symptoms. Anti-diabetic therapy could be de-escalated to a combination therapy with metformin and empagliflozin without insulin. Follow-up sonography was unremarkable for signs of inflammation as seen upon initial presentation (**Figure 3**).

## 3 Discussion

This is the first report of a patient with autoimmune pancreatitis successfully treated with vedolizumab. High-dose corticosteroids is the first line treatment, but a relapse of symptoms occurs in approximately 60% of patients with type 1 and less than 10% of patients with type 2 AIP when tapered (14). In this case, another taper may be attempted with the option for supportive immunosuppression and relapse prevention through azathioprine, mycophenolate mofetil, or 6-mercaptopurine. Rituximab may also be considered as an escalation therapy, although the evidence is stronger in IgG4 seropositivity (15). More specific treatment options will require a deeper understanding of the underlying pathophysiology.

Although AIP can be distinguished into subtype 1 and 2 clinically, this does not yet have any therapeutic consequences. This is partially because the classical division into “B cell -” and “T cell disease” is challenged by heterogenous observations both in mouse models and human cases: T cells may play a role in both subtypes, as polymorphisms in cytotoxic T lymphocyte-associated antigen 4 (CTLA-4) have been associated with higher risk of developing IgG4 positive AIP (16). T cell involvement has also been a proposed mechanism in a clinical case of type 1 pancreatitis (17). Additionally, effector T cell suppression and regulatory T cell expansion through cyclosporin A and rapamycin respectively have been proposed as treatment options in mouse models of AIP (18).

Rituximab is classically thought to work through reduction of antibodies produced by autoreactive B cells - which is why it is thought to help especially in IgG4 positive disease (15) - but there is evidence that rituximab might work through diverting macrophages away from inflammatory immune complexes (19). The complement system might contribute to disease by recognition of such immune complexes, with an unclear role for IgG4 - as it does not bind C1q - and possible involvement of IgG1-complexes (20).

The efficacy of Vedolizumab in this case does not necessarily function as an argument pro “T cell disease” since its mechanism is privy to further investigation itself. We have recently shown that α4β7-blockade does not change the percentage of lamina propria CD4+ and CD8+ T cells in patients, but that it has a substantial effect on the innate compartment, specifically Macrophages (21). Another group has reported a shift in balance within the CD4+ window toward regulatory function by preferentially excluding conventional T cells (22).

Since AIP is a rare disease, there are no definite guidelines for the even rarer case of treatment failure or unavailability of agents. Increasing expertise with novel biological agents, as well as advances in the understanding of pathophysiology could help in finding individual treatment options for such cases. Conversely, such individual approaches could aid in elucidating the underlying pathophysiology.

Here, we reviewed the current state of knowledge about mechanism of disease, expression patterns, and disease associations to rule in α_4_β_7_ integrin blockade as a new mode of action and potential treatment option for this rare inflammatory disease. We hope that our considerations and experience will help clinicians facing AIP treatment failure and will lead to further investigation both within experimental models and in the form of properly constituted clinical trials.

## 4 Patient’s perspective

“Searching for my symptoms online had me extremely worried about pancreatic cancer. So, I was very relieved when I got the diagnosis of autoimmune pancreatitis. The steroids quickly helped but left me continuously exhausted and limited in my day-to-day life. I just wanted to get rid of them. With vedolizumab I had to learn to take things easy for two days following the infusion, since I got tired quickly and developed headaches. This being an acceptable downside, I finally feel healthy again and can live as I did previously. I am glad my case is getting published, and I hope it may help doctors dealing with similar difficulties.”

## Data availability statement

The original contributions presented in the study are included in the article/Supplementary Material. Further inquiries can be directed to the corresponding author.

## Ethics statement

Ethical review and approval was not required for the study on human participants in accordance with the local legislation and institutional requirements. The patients/participants provided their written informed consent to participate in this study. Written informed consent was obtained from the individual(s) for the publication of any potentially identifiable images or data included in this article.

## Author contributions

PG: preparing manuscript (original draft). FT: preparing manuscript. JL and SS: patient management, providing diagnostic and treatment results. All authors contributed to the article and approved the submitted version.
